# Impact of Particles on Pulmonary Endothelial Cells

**DOI:** 10.3390/toxics10060312

**Published:** 2022-06-09

**Authors:** Marina Almeida-Silva, Jéssica Cardoso, Catarina Alemão, Sara Santos, Ana Monteiro, Vítor Manteigas, Ana Marques-Ramos

**Affiliations:** 1 HTRC-Health & Technology Research Center, ESTeSL—Escola Superior de Tecnologia da Saúde, Instituto Politécnico de Lisboa, 1990-096 Lisbon, Portugal; marina.silva@estesl.ipl.pt (M.A.-S.); jesscrds@hotmail.com (J.C.); catarinaalemao98@gmail.com (C.A.); sararitamartins@hotm.com (S.S.); ana.monteiro@estesl.ipl.pt (A.M.); vitor.manteigas@estesl.ipl.pt (V.M.); 2 Centro de Ciências e Tecnologias Nucleares (C2TN), Instituto Superior Técnico, Universidade de Lisboa, Estrada Nacional 10, ao Km 139.7, 2695-066 Bobadela-Loures, Portugal

**Keywords:** pulmonary endothelial cells, particles, impact, exposure, pollution

## Abstract

According to the WHO, air quality affects around 40 million people, contributing to around 21,000 premature deaths per year. Severe respiratory diseases, such as asthma and chronic obstructive pulmonary disorder, can be promoted by air pollution, which has already been documented; this is one of the reasons why air quality is a very relevant factor for human health and well-being. Aerosols are an aggregation of solid or liquid particles dispersed in the air and can be found in the form of dust or fumes. Aerosols can be easily inhaled or absorbed by the skin, which can lead to adverse health effects according to their sizes that range from the nanometre to the millimetre scale. Based on the PRISMA methodology and using the Rayyan QCRI platform, it was possible to assess more than four hundred research articles. This systematic review study aimed to understand the impact of particles on pulmonary endothelial cells, namely particulate matter in different sizes, cigarette smoke, diesel exhaust particles and carbon black. The main conclusions were that particles induce multiple health effects on endothelial cells, namely endothelial dysfunction, which can lead to apoptosis and necrosis, and it may also cause necroptosis in lung structure.

## 1. Introduction

Air quality has a significant influence in human health and well-being [1], and it has been recognised that air pollution contributes to severe respiratory diseases such as asthma and chronic obstructive pulmonary disorder (COPD) [2]. An aerosol is an aggregation of solid or liquid particles dispersed in the air, which can be found in the form of dust or fumes. Due to particle sizes ranging from the nanometre to the millimetre scale, these particles can be easily inhaled or absorbed by the skin, reaching the cells of the pulmonary alveoli and finally entering the bloodstream, which can lead to health adverse effects [3,4]. These include proinflammatory responses in vivo, such as the increased production of reactive oxygen species (ROS), the altered transcription of genes related to inflammation, the polarisation of macrophages and the overproduction of proinflammatory molecules [5]. Ultimately, be exposed to aerosol induces cardiovascular and respiratory diseases that, in some cases, might be fatal [1].

Air pollutants such as particulate matter (PM), polycyclic aromatic hydrocarbons (PAHs), carbon monoxide (CO), gaseous mixtures of nitrogen dioxide and volatile organic compounds can lead to severe lung damage and respiratory diseases such as asthma, bronchitis and chronic obstructive pulmonary disorder [2]. It is now known that the guidelines for air quality are not met in the 115 largest cities in the European Union (EU), which affects around 40 million people, ultimately leading to 21,000 premature deaths per year [6]. In Southeast Asia, it was estimated that in 2016, air pollution resulted in about 2.4 million premature deaths [6]. In other regions, in 2012, the number of deaths was also high: 194,000 deaths in the Eastern Mediterranean, 1.1 million in the Western Pacific and 211,000 deaths in Sub-Saharan Africa [7]. Accordingly, the study of the molecular mechanisms behind the effects of air pollutants on human health is mandatory. Among the most common and studied air pollutants are PM, cigarette smoke (CS), diesel exhaust particles (DEPs) and carbon black (CB).

Exposure to PM, specifically the particles derived from combustion, has increased significantly in Western countries in the last 250 years due to globalisation developments and industrialisation, such as increased traffic and shipping activities. This has led to the presence of substantial quantities of coarse (aerodynamic diameter < 10 μm—PM10), fine (aerodynamic diameter < 2.5 μm—PM2.5) and ultrafine (aerodynamic diameter < 0.1 μm—PM0.1) particles in the near-surface atmosphere [8]. Epidemiological and experimental studies have proven that exposure to atmospheric PM, especially fine particulate matter with sizes of 2.5 µm (PM2.5) or below, contributes to 3.3 million premature deaths per year worldwide [9], which are caused by respiratory and cardiovascular diseases such as ischemic heart disease, COPD, acute lower respiratory illness and lung cancer [5]. Studies have also shown that PM accelerates inflammation-mediated thrombosis [9]. It has been estimated that exposure to high concentrations of PM2.5 affects approximately 92% of the world’s population in areas with adverse air quality [1].

Nearly 1.25 billion people smoke cigarettes daily worldwide [10]. CS is a highly complex mixture that contains more than 4000 compounds, such as nicotine, a component of the tar phase that is the addictive substance of cigarette smoke [11]. Environmental tobacco smoke is generated from the combination of sidestream smoke (85%), smoke emanated from the burning part of a cigarette which contains a higher concentration of toxic gaseous constituents, and a small fraction of exhaled mainstream smoke (15%), which is smoke that is drawn through the tobacco into an active smoker’s mouth, which contains 8% tar and 92% gaseous components [11]. CS is linked to the most common causes of death in the elderly and contributes to the morbidity and disability rates associated with many chronic illnesses that are common in this age group [12].

Ambient air pollution by DEPs is a major cause of cardiovascular and metabolic mortality worldwide [13]. Diesel is a complex mixture of solid and liquid material with respirable size that can penetrate deep into human lungs [14]. DEPs combine approximately 20% ambient PM and belong to the fine (PM2.5) and ultrafine particle fractions (PM0.1); exposure to DEPs can impair endothelial and fibrinolytic functions, promote blood thrombogenicity and increase the gravity of cardiac ischemia in humans [13].

CB is a fine material that is used as a black pigment and is produced by the thermal decomposition of liquid or gaseous hydrocarbons under controlled conditions, which mainly consist of elemental carbon, with the rest being hydrogen, sulphur, oxygen, organic carbon, extractable organic materials and ash [15]. CB has high chemical stability and electrical conductivity and is a conductive additive [16]. Exposure to CB occurs in workers in industries that produce rubber and fortify vehicle tires and other rubber products, and it is used in coatings, plastics, inks or paints, ceramics, paper, battery production, carbon electrode production and in metallurgical processes such as carburisation [15]. In terms of the effects of CB on cells, it has been demonstrated that it strongly induces the production of reactive oxygen species (ROS) [17]. Additionally, studies on mice regarding gestation with CB through intratracheal instillation showed a change in testicular structure and reduced daily sperm production [18].

It has long been suggested in studies, in vitro and in vivo, that PM, CS, DEPs and CB can affect the endothelium and its cells, inducing lung injury and inflammation via endothelial dysfunction [13,19,20,21].

The endothelium is a layer that covers the interior of the walls of arteries, capillaries, veins and the lymphatic system [19]. This monolayer plays an active role by regulating the environment, responding to external stresses and also presenting itself as an endocrine and metabolic tissue [22]. Endothelial cells (ECs) guarantee permeability for the passage of blood, hormone fluids, macromolecules and platelets through arteries, veins, arterioles and venules and controls blood flow and vascular relaxation and constriction [23,24,25]. ECs secrete mediators and signalling molecules that regulate blood clotting to prevent platelet adhesion and aggregation in conditions of homeostasis [19]. The structure, characterisation and function of ECs vary according to their location [19]. Their structural heterogeneity includes differences in size, thickness, the position of the nuclei [19], changes in the genetic expression and in the production of the extracellular matrix and in the properties of the cell surface [25]. The function of pulmonary endothelial cells (PECs) is not very different from vascular endothelial cells; they regulate blood flow but also control the passage of liquid and macromolecules between the interstitial space, the vessels and the smooth muscle cells [26,27,28]. The role of PECs in haemostasis regulation can have opposite effects, as it displays anticoagulant, antiplatelet and fibrinolytic properties, whereas it promotes contrasting responses in the case of injury [29]. It also regulates the synthesis and metabolism of vasoactive compounds, which are regulators of pulmonary lung tone, such as endothelin-1 (ET-1) and nitrogen monoxide (NO) [30].

Based on the evidence that air particles are easily inhaled and deposited on the human respiratory tract, and knowing that their effects are poorly understood, we investigated the impact of the following pollutants on PECs: PM, CS, DEPs and CB.

## 2. Materials and Methods

This study includes data published between 1 January 2000 and 6 March 2021 regarding studies about the effects of aerosols on endothelial cells, following the PRISMA methodology. The search terms used for each platform are described in Table 1.

This search led to four hundred and eleven research articles in all databases, which were uploaded to an online and free platform named Rayyan QCRI. Through the application of the inclusion and exclusion criteria (Table 2), it was possible to select seventy-eight articles, of which seventeen were selected as eligible.

**Figure 1:**
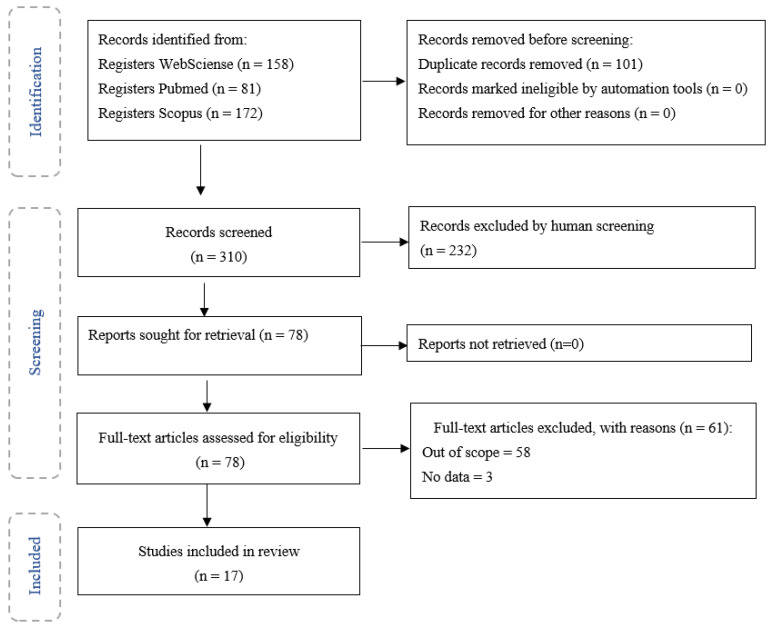
PRISMA methodology of selection of papers.

The diagram in Figure 1 describes the different phases of the selection of papers and the papers that were obtained in the final phase.

## 3. Results

From the seventy-eight articles obtained, seventeen were selected and analysed, being characterised according to the type of pollutant and its effects/impact on endothelial cells. The following air pollutants were found: CS, PM, including PM1, PM2.5, PM10, and fine and ultrafine nanoparticles, DEPs and CB (see Table 3). A total of nine articles assessed the effects of such pollutants on endothelial cells, through in vitro studies, using the following cells: lung endothelial cells in five articles, human pulmonary microvascular endothelial cells in three articles, and one article used rat microvascular cell monolayers. Three articles used in vivo methods, two with mice and one with humans. Finally, six articles that used both methods were analysed, with mice subjects used in (1) in vivo studies and (2) lung endothelial cells, lung microvascular endothelial cells, human pulmonary microvascular endothelial cells (HPMVECs), human pulmonary endothelial cells and pulmonary vascular endothelial cells in in vitro studies.

## 4. Discussion

**Figure 2:**
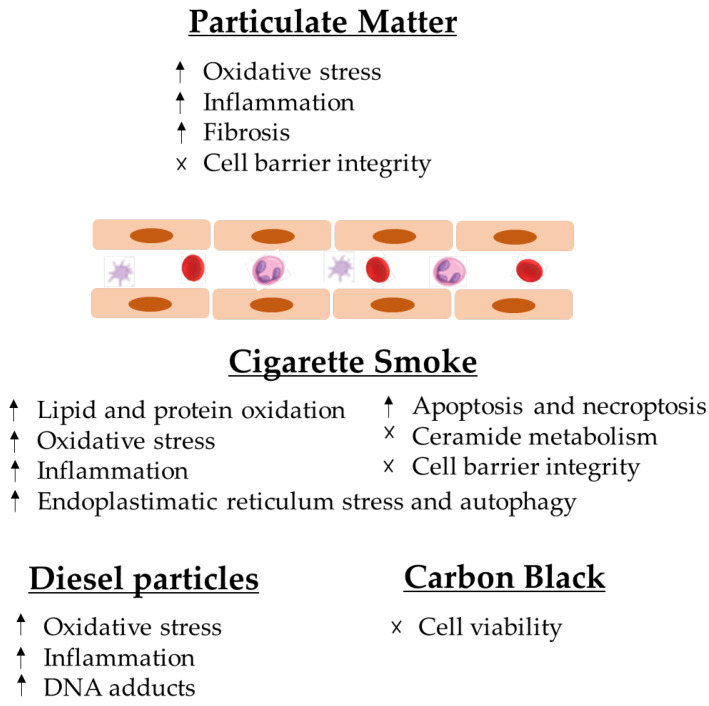
Effects of particulate matter and, consequently, cigarette smoke, diesel particles and carbon black on lung endothelial cells.

The analysed articles led the authors to conclude that particles induce multiple health effects on endothelial cells, namely endothelial dysfunction, which can cause apoptosis and necrosis, and it may also cause necroptosis in the lung structure (Figure 2).

### 4.1. Particulate Matter

Particulate matter (PM) can cause respiratory and cardiovascular diseases such as chronic obstructive pulmonary disease, ischemic heart disease, COPD, acute lower respiratory illness and lung cancer [5]. It affects around 40 million people, ultimately leading to 21,000 premature deaths per year [6]. Exposure to PM comes from combustion and can be presented in different sizes.

The results show that PM induces the disruption of the lung endothelial barrier through the induction of ROS and concomitant oxidative stress [31,37], with the activation of the p38 MAPK and HSP27 signalling pathways [37]. In particular, it was observed that the PM-induced phosphorylation of HSP27, an important protein in regulating actin polymerisation [47], leads to a contractile cytoskeletal arrangement [37] and that actin filament reorganisation was dependent on ROS production and p38 MAPK activation [37]. Accordingly, in order to protect endothelial integrity against PM exposure, it is necessary to restrain the activation of the p38–HSP27 pathway through the inhibition of oxidative stress [37]. Similarly, Karki, P. et al. [32] revealed that the effects of PM on EC barrier dysfunction were dependent on the production of ROS, which induced the production of truncated oxidised phospholipids (Tr-OxPLs). These events resulted in the destabilisation of cell junctions, as Tr-OxPLs induce the phosphorylation of the adherent junction VE-cadherin, which results in its internalisation with concomitant rescue from the plasma membrane [32]. Accordingly, the uncontrolled production of ROS seems to play a critical role in the PM-mediated effects on ECs. This event is caused by different mechanisms such as the activation of pro-oxidant enzymes and mitochondrial damages, and it constitutes a recognised mechanism of inflammation and cellular damage [32].

Endothelial cells are the main tight-junction-associated cellular barriers for pulmonary gas exchange, and normally, they are impermeable to albumin and leukocytes. The place of adhesion between leukocytes and endothelial cells is determined by the presence of molecules such as VCAM-1, a very important marker for leukocytes, including PMNs [34]. The data collected showed that PMNs treated with PM2.5 are able to bind sVCAM-1 and that PM2.5 exposure induces PMN infiltration with increased VCAM-1 [34]. Furthermore, it was demonstrated that short-term exposure to PM2.5 increases neutrophil count in the peripheral blood and the neutrophil to WBC ratio, which indicated that PMNs participate in PM2.5-induced inflammatory-lung injury. This hypothesis was confirmed when PM2.5-induced lung injury was reversed by the depletion of circulatory PMNs before PM2.5 exposure [34]. Furthermore, as PM2.5 deposits in the alveolar space and ECs, it causes lung oedema, morphological changes and cell barrier disruption [34]. When it comes to ultrafine PM, mainly PM1, the decrease in cells’ viability depends on the concentration and exposure time, and it has been demonstrated that PM1 exposure induces the release of the proinflammatory cytokines IL-6 and TNF-α by surrounding epithelial cells and macrophages [36]. This resulted in the upregulation of ICAM-1 on endothelial cells through the activation of TNF-α/NF-κB signalling pathways, which promotes the adhesion of monocytes to ECs, a key event in the initiation of atherosclerosis [36]. Similarly, Mo et al. [38] demonstrated that UFP exposure (10 and 20 μg/mL) resulted in IL-6 induction through the activation of p38 and ERK1/2 MAPKs. In this study, the authors also demonstrated that these effects are caused by the induction of ROS, which occurs in a NAPDPH-oxidase-dependent manner [38].

By using tri-co-cultured systems consisting of alveolar epithelial cells, ECs and macrophages, Wang et al. [33] demonstrated that PM2.5 exposure induces a potent inflammatory response. In this study, EA.hy926 endothelial cells were not exposed to particulate matter directly, but different doses were chosen to study the mechanistic effects on both the cardiovascular and pulmonary system. Based on this methodology, it was possible to determine that the basolateral medium LDH that represents the cytotoxicity of EA.hy926, after PM2.5 exposure, did not induce significant EA.hy926 cytotoxicity, but the LDH levels were elevated meaningfully in high doses of PM2.5-treated apical co-cultures [33]. No reason was found for the different levels of LDH [48], but is known that its release into the extracellular space is a result of compromised or damaged cell membrane [48]. However, Bengalli et al. [39] found that exposure to PM10 increased IL-1β, a cytokine responsible for inflammatory responses on the cell [49], in ECs on the basolateral compartment; yet, no release in the apical compartment was observed, and it was not possible to find an explanation for that. The ABB (air–blood barrier) treated with CuO did not show a response in the basolateral compartment; this could be due to the integrity of the ABB which can inhibit the passage of CuO in the direction of the endothelial cells, while it enables to rise IL-6 and IL-8 release from the apical compartment [39]. Bengalli et al. [39] suggested that an explanation for CuO being free in the cytoplasm might be its higher oxidative potential compared with TiO2, and this can cause an oxidative degradation of lipids in vesicles, leaving CuO free. Exposure to PM10 and nanoparticles was shown to induce modulation in proinflammatory proteins and ECs [39].

In another study by Wang. et al. [37], an increase in Akt phosphorylation in endothelial cells due to the presence of NaSH was also reported, being dependent on the dose and the time of the exposure. This phosphorylation could be inhibited through the inhibition of PI3K in the presence of its inhibitors, LY 294002, leading Wang, T. et al. [37] to suggest that H_2_S stimulates Akt activation and that NaSH prevents not only vascular hyperpermeability through the system that combines ROS cleaning and Akt activation, but also endothelial integrity, thus suggesting that H_2_S may attenuate pulmonary inflammation caused by PM, although the associated mechanisms are still unclear [37].

Wang et al. [33] also showed that some inhaled PM2.5 can cross the epithelial barrier to the endothelial cells, causing damage in the cells, which leads to morbidity and mortality caused by cardiovascular diseases.

Ma et al. [35] reported that transforming growth factor-β (TGF-β) is decisive in the processes of development, injury and restoration in the lung tissue of mice. In this article, it was shown that exposure to PM2.5 leads to an activation of the TGF-β1 receptor that plays a fundamental role in the pathological process of pulmonary fibrosis, promoting ENDMT in the MHC cells, increasing the levels of Col1 and Acta2 and reducing the expression of CD31 and CDH5 after 48 h. After quantitative reverse transcription PCR (RT-qPCR) analysis, the lncRNA Gm16410 gained attention, because it is known that lncRNA Gm16410 is a pseudo-repressor gene of STAT interaction and it has been described that STAT3 and TGF-β1 form a closed loop, which can raise fibrosis in lung cancer cells [38].

#### 4.1.1. Cigarette Smoke

It is established that CS interferes with the capacity of airway epithelial cells to uphold repair processes and also can lead to modifications in the airway structures and functions that characterise COPD [10]. It can cause detrimental cell responses, inducing tissue damage around the alveoli and airways, with the subjacent development of various lung diseases [50], such as lung cancer and asthma (9). From a mechanistic point of view, it has been demonstrated that CS increases the risk of lipid and protein oxidation and deregulates the normal ceramide metabolism, endoplasmic reticulum (ER) stress and cell death [50] (Figure 2). Furthermore, it has been reported that it can induce xenobiotic and redox-regulating genes and numerous oncogenes, whereas it blocks the expression genes that regulate airway inflammation as well as diverse tumour-suppressor genes [10] (Figure 2).

One of the molecular effects of e-smoking is the reduction in NO levels [40], and it is known that NO loss is a key event for endothelial dysfunction [51,52]. In this regard, it has been demonstrated that HPMVEC subjected to serum from regular e-cigarette smokers produce ROS and increase ICAM-1 expression, demonstrating endothelial activation and inflammation, which together represent the first step in signalling events that are conducive to the pathogenesis of numerous vascular disorders [40]. It seems that the activation of NADPH oxidase 2 is the origin of ROS production, as a decrease in ROS production following the pre-treatment of HPMVECs with the NADPH oxidase 2 inhibitor—apocynin—was observed [40]. Furthermore, it is known that NO reacts with ROS to create peroxynitrite, which triggers a cellular response that can lead to apoptosis and necrosis [48,49]. Indeed, it is known that CS induces the death of epithelial and endothelial lung cells through apoptosis [53,54]. Actually, both cultured and mouse-lung ECs exposed to CS exhibit pronounced apoptosis, which is accompanied by emphysema [42]. The mechanisms that seem to induce these effects are the activation of endoplasmic reticulum (ER) stress and autophagy with increased eIF2α signalling. Additionally, in those settings, FAK signalling, a prosurvival pathway, is inhibited through oxidative stress [42]. It is noteworthy to emphasise that the author points out that the induction of the unfolded protein response (UPR) (a response to ER stress) is not an injury but rather a compensatory mechanism to protect from lung EC injury through the induction of the apoptotic death of dysfunctional cells [42]. This suggestion was based on the observation that the evolution of lung EC apoptosis and emphysema was associated with the impairment of eIF2α signalling, which occurs when UPR is diminished [42].

The mechanism by which CS induces cell death seems not to be restricted to apoptosis, as Mizumura, K. et al. [41,53] revealed that chronic CS also induces the mitophagy of lung structural cells that ultimately leads to necroptosis, a caspase-independent form of regulated cell death [41,53]. This event occurs through the induction of acid sphingomyelinase and the accumulation of the ceramide C16-Cer in a PINK1-dependent manner [41]. It is known that both apoptosis and necroptosis might contribute to the pathogenesis of COPD, particularly to its emphysema phenotype.

Epithelial and ECs form an important barrier in the alveolus, forming a semi-permeable interface for gas exchange that prevents alveolar and interstitial oedema formation [55,56]. A study by Schweitzer, K.S. et al. [43] demonstrated that CS exposure induces the disruption of the endothelial cell barrier. From a mechanistic point of view, CS induces the upregulation of neutral sphingomyelinase-mediated ceramide and p38 MAPK and JNK activation in addition to oxidative stress and Rho activation. Neutral sphingomyelinase activation is responsible for the observed morphological changes upon CS exposure, as it induces actin cytoskeletal rearrangement, junctional protein zonula occludens-1 loss and intercellular gap formation [43]. Schweitzer et al. [43] referred to the importance of the development of CS-induced emphysema because it is believed that oxidative stress and ceramides, which also have a role in regulating apoptosis [57], are involved in the emphysema pathogenesis. This mechanism may make endothelial cells more sensible to effects of subsequent injuries associated with ARDS which can evolve to chronic inflammatory changes associated with COPD [43].

#### 4.1.2. Diesel Particles

As cited before, DEPs are a mixture of solid and liquid material that easily penetrate human lungs [14], being able to damage endothelial and fibrinolytic functions, stimulate blood thrombogenicity and intensify the gravity of cardiac ischemia in humans [58]. Regarding diesel particles, which are an ensemble of compounds, extremely dependent on fuel and engine, and have physical–chemical characteristics, studies about their effects often use standard reference materials (SRMs), and according to the results, DEPs are constituted of ultrafine particles and a tendency toward aggregation [44]. Regarding the DEPs tested by Bengalli et al. (2017) [45], SRMs were developed under controlled conditions (SRM, 1650b), being related to the emission of a heavy engine and a light engine (SRM, 2975). The chemical speciation of the EuroIV (light duty engine—types of vehicles that are under the emission limit, defined in Directive 98/69/EC) revealed a common PAH composition with elevated levels of phenanthrene, pyrene, dibenzo(a,h) anthracene and benzo[a]anthracene [44]. A series of events that linked UFP exposure to inflammation and oxidative stress with the consequential indirect activation of the vascular endothelium [44] and the release of VEGF from BEAS-2B [44] has been demonstrated. The risk of exposure on endothelial cells by PAHs is related to the fact that due to its activation, it can affect DNA, forming DNA adducts, leading to deletions, translocations, fusions or aneuploidy [59] through the formation of phenols, catechols, diol-epoxides, quinones, o-quinones and radical cations, which occurs during that formation [60,61,62].

The availability of IL-6R in BEAS-2B supernatants was assessed, revealing its overexpression after diesel exposure [45]. With the exposure of EURO IV, an increased expression of ICAM-1 and VCAM-1 was also visible, and also, the release of proinflammatory mediators (IL-6), which was also reported by Bengalli et al. [45]. The diesel-induced overexpression of ICAM-1 and VCAM-1 was successfully inhibited by interfering with IL-6/gp130 binding by the addition of an IL-6 antibody to epithelial cell media to reveal the important role of IL-6 released by epithelial cells in inducing the observed effects on endothelial cells [45].

Meanwhile, by Klein et al. [8], it was demonstrated that DEPM exposure did not induce the expression of relevant markers for endothelial inflammation such as ICAM-1 or E-selectin, important for the adhesion of inflammatory cells and the induction of inflammation, leading to diseases such as myocardial infarction and atherosclerosis [8]. After exposure for 24 h and 48 h, the increase in the expression levels of the transcription factor NFkB (known to upregulate cytokines and the transcription of cellular adhesion molecules in the beginning of inflammation) and the chaperone HSP70 (a general marker for cellular stress) point towards a delayed response after acute exposure to DEPM [8]; these findings may indicate endothelial dysfunction and cardiovascular disease [8]. Nevertheless, this led to high nuclear translocation of the transcription factor Nrf2 and the expression of CYP1A1 mRNA in endothelial cells. The cellular viability or inflammatory statuses were not affected by the applied doses of DEPM [8]. In addition, in DEPM, the expression of HSP70 can be found, which was upregulated to respond to PAHs, such as B[a]P [8]. In addition, in the upregulation of CASP7 mRNA, after being directly exposed to DEPM, there was a delayed stress response in the endothelial cells that led to an upregulation of the marker genes and also indirect exposure to tetracultures in the endothelium after being exposed for 48 h [8].

In research by Bengalli et al. (2017) [45], it was demonstrated that cells that suffered indirect exposure to DEP had an overexpression of ICAM-1 and VCAM-1, and once directly exposed, IL-6R levels would also increase [45]. IL-6 is released on site in the case of inflammation [63]. IL-6 trans-signalling was also reported to increase the expression of molecules in endothelial cells [64]. Contributions to endothelial activation and proangiogenic processes are made by CAM-1 and ICAM-1, which can be induced by vascular endothelial growth factor (VEGF) and cytokines [65].

Through the interaction with endothelial cell membranes and FLT1, KDR or NRP1 receptors and the subsequent activation of RAS and MAPK kinases signalling cascades, VEGF is a powerful inducer of angiogenesis [45], which can have a very important role in cardiovascular diseases. In the study by Bengalli et al. [45], it was found that angiogenesis-related proteins in endothelial cells exposed to conditioned media and the modulation of the MAPK pathway was weak, so it was assumed that this was due to a low level of VEGF, and it is known that only higher levels can have effects on endothelial cells [45].

The study by Bengalli et al. (2017) [45] demonstrates that modulated genes which are oxidative-stress-responsive include NFE2L2 transcription factor and its targets TXNRD1 and HMOX1, enzymes that protect against oxidative stress. Amid genes with altered expression, some were related to the MAPK pathway, which influences signals from different stimuli and responds appropriately in cells with responses, such as cellular proliferation, differentiation, development, inflammatory responses and apoptosis [66]. By increasing the expression of phosphorylated active forms, specific Akt1/2 and ERK2, identified to respond to growth factor signals, it was possible to validate the pathway activation. It was also shown that these proteins are activated by ROS and are involved in the regulation of various processes including cytokine production, metabolism, proliferation, angiogenesis, growth and cell survival [45]. It has been reported that NF-kB is activated by Akt, a renowned factor inducing cytokine transcription, which through NF-kB and STAT3 regulation is able to directly modulate IL-6 levels. It was confirmed that lung epithelial cells exposed to diesel particles released IL-6 [45].

#### 4.1.3. Carbon Black

Finally, only one study selected tried to identify the relation between carbon black and its effects on endothelial cells. As previously stated, CB is mainly used as a black pigment, and individuals’ exposure to it occurs in industries. Based on Dinmohammadi et al. [46], the interaction poorly contributes to prothrombotic and proinflammatory effects during the cells’ exposure, as shown by unclear evidence. As it can be seen in Table 2, Dinmohammadi et al. [46] did not find changes in FVIII, VWF, P-selectin and IL-8 release when cells were exposed for 24h to CB or MWCNTs at lower doses (under 10 µg/mL), while treatment with 100 µg/mL showed a decrease in cell viability.

Both FVIII and VWF are storage proteins in the endothelium which are released once stimulated by other exogenous or endogenous molecules [46], mediating platelet adhesion (Peyvandi et al., 2011), which binds to injured areas (Packham et al., 1984). FVIII has a role in the coagulation cascade [67] and is synthetised in endothelial cells [64]; the authors previously observed an increase in FVIII in cardiac microvascular endothelial cells once exposed to UFPs and measured the same protein in HPMECs once exposed to CB, but no results were visible. VWF is also a protein that can be found in endothelial cells, linked to haemostasis, by binding to FVIII [68]. The protein P-selectin can be also found on the surface of platelets and endothelial cells once it is activated [69], influencing the regulation of platelet function [70]. Finally, IL-8, which is a cytokine which is produced by different cells and is linked with inflammation [71], has been associated with a direct role in the survival, proliferation and angiogenesis of endothelial cells [72].

These findings showed that no changes occurred in Weibel–Palade bodies (WPBs), which store all these proteins [73], are involved in inflammation [68,74,75] as inflammation mediators and are also linked to coagulation factors [73]. Since there was no response from endothelial cells to the UFPs and no increases in these proteins, this study [46] concluded that exposure did not contribute to prothrombotic and proinflammatory effects.

The analysed articles lead us to conclude that particles induce multiple effects on endothelial cells, namely endothelial dysfunction, which can lead to apoptosis and necrosis, and it may also cause necroptosis in lung structures (Figure 2). Several studies have been published that have relevant results for the scientific community regarding exposure to atmospheric particles. Barosova et al. [76] studied the impact of nanomaterials in 3D lung co-cultures, realising that acute or prolonged exposure to nanomaterials may induce different responses [76]. Offer et al. [77] also used an air–liquid interface to assess lung cells’ exposure to particles and demonstrated the importance of the chemical PM composition in adverse health outcomes [77]. This evidence is in line with the most recent articles in this field [76,77,78,79].

## 5. Conclusions

The main conclusions of this systematic review are outlined in Figure 2.

Particles can cause inflammation in cells, the formation of paracellular gaps, cytoskeleton rearrangement, barrier dysfunction, emphysema and increase ICAM-1 and ROS generation, which contribute to the pathogenesis of many vascular disorders, acute respiratory distress syndrome and chronic obstructive pulmonary disease. Exposure to aerosols may contribute to asthma, cardiac arrhythmias, myocardial infarction and atherosclerosis.

Further investigations are suggested to create: (1) more knowledge about the impact of carbon black on endothelial cells; (2) as well as knowledge about the PM2.5 mechanism on apical and basolateral membranes; and (3) practical studies on inhibitory pathways to reduce the effects on endothelial cells from aerosols.

Several limitations were identified throughout this systematic review: (1) a high number of articles related to air pollution, but few specified the pollutant linked with the impacts on endothelial cells; (2) the authors had to exclude a massive number of articles since silica particles, nanoplastics or other type of pollutants that were not particles were used, as well as other types of cells; (3) the methodologies used in the different articles were not exactly the same, which means that the comparison and integration of the information have associated limitations, although we tried to account for these differences.

## Figures and Tables

**Table 1:** Description of search databases and search terms.

Search Databases	Search Terms
Pubmed	“endothelial cells” [MeSH Terms] AND “lung” [MeSH Terms] AND (“particulate matter” [MeSH Terms] OR “particle*” [All Fields])
Web of Science	“endothelial cells” AND “lung” AND (“particulate matter” OR “particle*”)
Scopus	“endothelial cells” AND “lung” AND (“particulate matter” OR “particle*”)

**Table 2:** Inclusion and exclusion criteria.

Inclusion Criteria	Exclusion Criteria
Articles in English language	Articles in other languages
Articles published from 1 January 2000 to 6 March 2021	Articles published prior to 2000
Articles related to lung endothelial cells	Articles related to other cells
Articles related to particulate matter effects	Articles related to another organs
Articles on in vitro and/or in vivo studies	Articles related with other pollutants

**Table 3:** Data obtained from the chosen articles.

	Pollutant	Objective	Methodology	Results	Main Findings
[31]	PM	The importance of the protective role of hydrogen sulphide (H_2_S) on PM-induced human lung EC barrier disruption and pulmonary inflammation.	(In vitro) HLMVECs. (In vivo) Mice.	− NaSH (pre-treatment) significantly attenuated PM-induced endothelial barrier disruption (50%) inhibition; − NaSH (pre-treatment) abolished the PM-induced, substantial production of human lung microvascular endothelial cells (HLMVEC ROS); − The addition of LY294002 to the HLMVECs significantly reduced the protective ability of NaSH against PM-induced EC barrier disruption.	− The study suggests that the role of H_2_S may involve the attenuation of ROS-mediated pulmonary inflammation by PM air pollution.
[32]	PM	To investigate if the PM challenge triggers the production of bioactive Tr-OxPLs by pulmonary EC.	(In vitro) Human pulmonary endothelial cells. (In vivo) PM administered as suspension in saline to mice.	− PM caused the redistribution of adherent junction proteins vascular endothelial cadherin (VE-cadherin) and p120-catenin from cell membrane fractions to cytosolic fractions; − The inhibition of ROS production with NAC attenuated PM-induced decrease in TER; − Tr-OxPLs in EC exposed to PM: increase in 1-Palmitoyl-2-(5-Oxovaleroyl)-sn-Glycero-Phosphocholine (POVPC), 1-Palmitoyl-2-Glutaroyl-sn-Glycero-Phosphocholine (PGPC) and Lysophosphatidyl Choline (lyso-PC) variants; − Intracellular type 2 platelet-activating factor acetylhydrolase (PAFAH2) overexpression attenuated PM-induced vascular endothelial cadherin (VE-cadherin) tyrosine phosphorylation and PM-induced endothelial permeability.	− Effects of PM on endothelial cells depend on the elevation of ROS, which triggers the production of barrier-disruptive Tr-OxPLs including POVPC, PGPC and lyso-PC; the PAFAH2-mediated inhibition of Tr-OxPLs production as a potential therapeutic approach to alleviate PM-induced complications in lung injury.
[33]	PM2.5	The stabilisation of a suitable in vitro model to investigate PM_2.5_-mediated toxicity in vascular endothelial cells.	(In vitro) The Transwell culture method was used on A549 cells in apical chamber and EA.hy926 cells in the basolateral chamber to establish bi-culture, while tri-culture systems consisting of co-culture (A549 cells and THP-1-differentiated macrophages) in the apical chamber and also EA.hy926 cells in the basolateral chamber.	− Three doses (20, 60 and 180 μg/mL) were used to study the mechanism effects on both the pulmonary and cardiovascular system; − PM2.5 exposure did not induce significant EA.hy926 cytotoxicity in basolateral medium; − LDH levels were elevated significantly in high doses of PM2.5-treated apical co-cultures.	− Toxic effects induced by PM2.5 are not restricted to epithelial cells but can be transferred into the endothelium. The tri-culture system will contribute to the explanation of the relation between PM2.5 and cardiopulmonary diseases.
[34]	PM2.5	To determine if polymorphonuclear leukocyte PMNs exacerbate PM2.5-induced lung in-jury and the role of vascular cell adhesion molecule 1 (VCAM-1) in this process.	(In vivo) The association between blood PMNs and ambient PM2.5 levels on the previous day was analysed. Neutropenia was achieved by injecting mice with PMN-specific antibodies. The inhibition of PMN infiltration was achieved by pre-treating PMNs with soluble VCAM-1.	− In the PMN-endothelial cell co-culture system, PM2.5 induced the markedly higher expression of VCAM-1 mRNA and protein. In contrast, soluble VCAM-1-pretreated PMNs attenuated PM2.5-induced VCAM-1 mRNA and protein expression; the treatment of PMNs with PM2.5 further significantly increased the permeability, which can be completely blocked by soluble VCAM-1 pre-treatment.	− VCAM-1-mediated PMN infiltration was essential for a detrimental cycle of PM2.5-induced inflammation and lung injury. The results suggest that drugs that inhibit PMN function might prevent the acute deterioration of chronic pulmonary and cardiovascular diseases triggered by PM2.5.
[35]	PM2.5	A simulation of the state of PM2.5 in a real environment and chronic exposure to PM2.5 in mice was used to construct a lung injury model to detect whether exposure to PM2.5 could trigger EndMT in the lungs.	(In vivo) Balb/c Mice. (In vitro) Pulmonary vascular endothelial cells (PVECs). The toxicity of PM2.5 investigated by determining its effect on the growth of MHC cells using the MTT assay.	− (In vivo) At the gene level, PM2.5 induced an increase in COL1α1 and Acta2, showing that chronic exposure to PM2.5 can induce EndMT in Balb/c lungs. − (In vitro) − PM2.5 exposure increased cell viability significantly and cell migration increased remarkably after 48 h of exposure; − PM2.5 decreased the expression of CDH5 and CD31 significantly in contrast; − PM2.5 enhanced the expression of Acta2 and COL1α1 (gene level); − PM2.5 induces EndMT in vivo and vitro.	− PM_2.5_ induces EndMT by regulating the TGF- β1/Smad3/p-Smad3 pathway. The involvement of lncRNA Gm16410 in PM2.5-induced EndMT highlights the potential of lncRNAs to promote pulmonary fibrosis under environmental pollution.
[36]	PM1	The examination of PM1-induced inflammatory response in the respiratory system and the indirect effect on endothelial cell function.	(In vitro) Transwell co-culture model of EA.hy926 cells with BEAS-2B cells and macrophages.	− RT-PCR results showed that PM1 could significantly upregulate the expression of Interleukin 6 (IL-6) in BEAS2B cells. − The levels of IL-6 and tumour necrosis factor α (TNF-α) significantly increased after BEAS-2B cells and macrophages were exposed to 12.5, 25, 50 and 100μg/mL PM1 for 12 h, while the IL-1β level first increased and then decreased; − PM_1_ regulated intercellular adhesion molecule-1 (ICAM-1) expression by promoting tumour necrosis factor α (TNF-α) and IL-6 release from macrophages and BEAS-2B cells.	− PM1 exposure markedly impacted the viability of BEAS-2B cells; BEAS-2B cells were exposed to PM1 - the mRNA expression level of IL-6 in BEAS-2B increased significantly; PM1 induced the expression of ICAM-1 in EA.hy926 cells by promoting the release of IL-6 and TNF-α from BEAS2B cells.
[37]	Fine/ultrafine PM	To analyse if PM, via enhanced oxidative stress, disrupts lung endothelial cells’ (LECs’) barrier integrity, thereby enhancing organ dysfunction.	(In vitro) Cultivated human pulmonary artery ECs on gold electrodes and assessed TER. The investigation of the involvement of ROS and mitogen-activated protein kinase (MAPK) signalling pathways.	− PM induces EC barrier disruption, which is partially determined by on ROS generation; − PM activates p38 MAPK target and Ser/Thr kinase MAPKAP2; − A reduction in the expression of the p38 MAPK β isoform significantly inhibited PM-induced barrier disruption; − Reductions in HSP27 expression (siRNA) reduced PM-induced EC barrier disruption; − PM challenge (10–100 mg/mL, 1h) increased F-actin fluorescence, and increased actin organisation resulted in the formation of paracellular gaps.	− These results demonstrate that PM induces ROS generation in the human lung endothelium, resulting in oxidative-stress-mediated EC barrier disruption via the p38 MAPK- and HSP27-dependent pathway. These findings support a novel mechanism for PM-induced lung dysfunction and adverse cardiopulmonary outcomes.
[38]	Ultrafine particles	To test if exposure to UFPs leads to endothelial cell O_2_ U−generation via NADPH oxidase and results in activation of endothelial cells.	(In vivo) Mice. (In vitro) MPMVEC.	− Significant cytotoxicity was observed when MPMVECs were exposed to 100 and 200 μg/mL UFPs (24 h); − After the exposure of MPMVECs to non-toxic doses (0–100 μg/mL) of UFPs (6 h–12 h), DCF fluorescence increased progressively; − ROS generation was found once mouse lungs were exposed to UFPs; − The increased phosphorylation of p38 and ERK1/2 MAPKs was found after the exposure of MPMVECs (0–50 μg/mL) to UFPs (1–6 h); − After 10 and 20 μg/mL UFP treatment for 12 h, the expression of IL-6 increased in MPMVECs.	− Exposure to UFPs MPMVEC ROS generation and NADPH oxidase are the main source of this ROS. The UFP-induced IL-6 upregulation was significantly inhibited by SB203580, demonstrating that IL-6 overexpression after UFP treatment occurs through the activation of p38. − Exposure to UFPs caused the activation of MAP kinases due to oxidative stress in endothelial cells, which resulted in the upregulation of IL-6 and may cause lung and systemic inflammation.
[39]	Nanoparticles/PM_10_	To report the effects of metal oxide NPs (CuO and TiO2) and of PM on an in vitro model of the ABB constituted by the type II epithelial cell line (NCI-H441) and the endothelial one (HPMECST1.6R).	(In vitro) HPMECs.	− PM_10_ induced an increase in IL-1β release from the basolateral compartment, while the apical compartment did not release any IL-1β; − ABB treated with CuO showed an increase in IL-6 and in Interleukin 8 (IL-8) release from the apical compartment; − Cells exposed to CuO and TiO_2_ showed the internalisation of CuO and TiO_2_ NPs; − CuO was found free in the cytoplasm, and nTiO_2_ was compartmentalised into vesicles.	− The results demonstrate that the apical exposure of alveolar cells induces the significant modulation of proinflammatory proteins also in endothelial cells.
[40]	E-cigarette smoke	The evaluation of the acute response to aerosol inhalation of e-cigarettes in terms of oxidative stress and indices of endothelial activation in human pulmonary microvascular endothelial cells (HPMVECs).	(In vivo) Healthy subjects were subjected to e-cig challenge. Serum was monitored for markers of inflammation (C-reactive protein (CRP) and soluble intercellular adhesion molecule (sICAM)) and nitric oxide metabolites (NOx).	− Serum CRP levels increased between 60 and 120 min but decreased thereafter; − Regular e-smoking individuals showed an average (baseline) serum NOx value of 16.6 ± 6.3 mol/L; − HPMVECs exposed to serum from regular e-cig smokers produced ROS comparable with smoking-naïve subjects exposed to e-cig inhalation.	− Acute e-cig aerosol inhalation was found to lead to a significant increase in oxidative stress and inflammation indices in the serum; − The Nicotinamide Adenine Dinucleotide Phosphate (NADPH) oxidase 2 pathway was key to ROS production by endothelial cells incubated with serum; − The effect of e-cig flavourings alone was reported to cause endothelial oxidative stress, although the source of ROS production was not identified.
[41]	Cigarette smoke	The investigation of the role of ceramide (Cer) in CS-induced lung cell mitophagy and cell death.	(In vitro) Cer and DHC treatments and aqueous CS extract (100%). (In vivo) DBA2/Jmice exposed for 5 h/d, 5 d/ wk, for 4 mo; PTEN-induced kinase 1 (Pink1)-/- mice exposed to room air or CS for 2 h/d, 5 d/wk, for 6 mo; CerS2-/- exposed to room air or CS for 2 h/d, 5 d/wk, for 1 mo.	− EC increased LC3BII when exposed to CS extract; − CS decreased p62; − CS-induced EC autophagy was associated with significant increases in C16- and C24-DHC and C16-Cer but not C24-Cer; − C16-Cer increases induced lung endothelial apoptosis; − CS exposure increased Cer in lung structural cells.	− C16-Cer induced mitophagy-mediated necroptosis in lung structural cells; − An increase was observed in C16-Cer levels in pulmonary epithelial and endothelial cells exposed to CS; − C16-Cer produced via ASM is an upstream initiator of PINK1-regulated lethal mitophagy by necroptosis in CS-exposed lung structural cells.
[42]	Cigarette smoke	A comparation between Focal Adhesion Kinase (FAK) activation in the lungs of highly and mildly susceptible mice after exposure to CS for three weeks.	(In vitro) Rat lung microvascular endothelial cells (LMVECs). (In vivo) Male C57BL/6 and FAK mice exposed to room air or CS for 3 weeks at 6 h per day and 4 days per week.	− Three weeks of CS exposure increased BAL inflammatory cells and elevated lung EC apoptosis in AKR mice; − LMVEC exposed to Cigarette Smoke Extract (CSE) for 6–24 h displayed an increase in apoptosis; − CSE decreased procaspase-9 levels 24 h after CSE exposure; − The restriction of FAK is associated with CS-induced lung EC apoptosis and the onset of early emphysema; − Three weeks of CS exposure in lungs of mice reduced phosphorylated eIF2α (Eukaryotic initiation factor 2 (alpha)), emphysematous lungs of AKR mice; − CSE enhanced the conversion of LC3B-I to LC3B-II.	− Lung EC apoptosis is increased in AKR mice; − FAK and eIF2α were inhibited in the lungs of susceptible AKR mice when early emphysema was evident upon CS exposure; − Diminished eIF2α and FAK signalling potentiates the apoptosis of lung EC induced by CS and the early onset of emphysema.
[43]	Cigarette smoke	The investigation of the occurrence and mechanisms by which soluble components of mainstream CS disrupt the lung endothelial cell barrier function.	(In vitro) Cultured primary rat microvascular cell monolayers.	− Treatment with N-acetyl cysteine (NAC) protected the endothelial barrier function in cells exposed to CS; − ECs treated with the caspase inhibitor exhibited a not constant protection from CS-induced permeability during the first hours of CS exposure; − The inhibition of neutral Sphingomyelinase (SMase) with GW4869 significantly attenuated CS-induced barrier dysfunction; − High and toxic concentrations of CS caused the dissolution of actin cytoskeleton; − Ceramide produced during CS exposure may be sufficient to induce barrier dysfunction through Rho kinase activation mechanism.	− Data suggest that CS induces subtle changes in the alveolo-capillary barrier directly, via oxidative-stress-dependent and ceramide-mediated cytoskeletal changes. This mechanism may render endothelial cells more susceptible to edemogenic effects of subsequent injuries associated with acute respiratory distress syndrome (ARDS) and may independently contribute, over time, to chronic inflammatory changes associated with COPD.
[44]	Diesel exhaust particles	The investigation of LECs’ primary response on bronchial cells BEAS-2B exposed to three different DEPs.	(In vitro) Microvascular lung HPMEC cells and BEAS-2B placed in aco-culture model and conditioned media.	− DEPs have a tendency to form bigger aggregates with regard to DEP 2975 and EuroIV; − DEP properties may affect damage and oxidative response.	− Air quality could increase with the depletion of the particle emissions to the environment, which could also result in the improvement in living conditions and lower exposure, mainly for people with a predisposition to develop CDV.
[8]	Diesel exhaust particulate matter (DEPM)	Researching the response of ECs to DEPM, using a complex 3D tetraculture model of the alveolar barrier.	(In vitro) Aerosol exposure of DEPM; viability evaluated at 6, 24 and 48 h after exposure.	− Increased HSP70 mRNA levels after 24 h and 48 h of indirect exposure; − Tetraculture did not result in augmented FAS mRNA levels after the indirect exposure to 80 and 240 ng/cm^2^; − CASP7 mRNA was significantly upregulated in endothelial cells that were indirectly exposed to 240 ng/cm^2^ at 48 h after the exposure; − The augmented expression of CYP1A1 after 6 h of exposure to 80 ng/cm^2^ of DEPM.	− DEPM exposure resulted in the activation and nuclear translocation of the transcription factor Nrf2 in endothelial cells.
[45]	Diesel UFP	The investigation of the endothelial cells’ activation through epithelial-released mediators’ role.	(in vitro) HPMEC-ST1.6R (EC) treated with 40% of media (epithelial cells diluted in M199 medium), HPMEC-ST1.6R cells exposure lasted for 24 h.	− The analysis of MAPK and angiogenesis pathways’ activation showed very weak modulation; − HPMEC-ST1.6R cells were indirectly exposed to DS particles: ICAM-1 and VCAM-1 protein expression; − Proteins (ICAM-1 and VCAM-1) in HPMEC-ST1.6R cells indirectly exposed to DS particles revealed an increase in its protein expression; − In cells exposed to diesel UFPs, IL-6R significantly increased; − ICAM-1 and VCAM-1 overexpression was reduced with the addition of IL and in samples exposed to diesel-conditioned media.	− In endothelial cells exposed to conditioned media, the weak modulation of the MAPK pathway and angiogenesis-related proteins was found; − In endothelial cells exposed to conditioned media, an increase in adhesitoxicityon molecules ICAM-1 and VCAM-1 was observed.
[46]	Carbon black (CB) and multi-walled carbon nanotubes (MWCNTs)	The investigation of industrial CB and CNTs’ potential toxicity in endothelial cells by measuring Coagulation factor VIII (FVIII) and the Von-Willebrand factor (VWF).	(In vitro) MECs, 48 h after reaching confluence, were treated with culture medium of either MWCNTs or CB with a solution of 0.1, 1, 10, or 100 μg/mL in.	− FVIII did not suffer significant changes: when HPMECs were exposed to 0.1, 1 and 10 μg/mL of MWCNTs or CB, it was possible to observe C, VWF: Ag, P-selectin or IL-8 levels; − The assay of cell toxicity showed that the treatment of ECs with 100 μg/mL MWCNTs for 24 h resulted in a 25% reduction in cell viability; − It was suggested that endothelial WPBs in the exposed cells and endothelial cell activation were not altered by UFPs.	− The cell viability assay did not demonstrate that CB exposure was toxic but did for EC exposure to 10–100 μg/mL; − The absence of endothelial cell response to UFPs demonstrates that in humans, direct carbon–EC interactions hardly contribute to proinflammatory and prothrombotic effects during exposure to ambient UFPs.
